# Moderating effects of academic involvement in web-based learning management system success: A multigroup analysis

**DOI:** 10.1016/j.heliyon.2021.e07000

**Published:** 2021-05-07

**Authors:** Sangeeta Mehrolia, Subburaj Alagarsamy, M. Indhu Sabari

**Affiliations:** School of Business and Management, Christ University, Bangalore, India

**Keywords:** Higher education, IS success model, Moderation analysis, Student involvement, Web-based learning management system, Structural equation modelling

## Abstract

While several educational institutions in India, in accordance to global practices, have adopted Web-Based Learning Management Systems (WLMS) to supplement classroom courses, it is largely seen that these WLMSs fail in their objectives, leading to little or no return on investments. The study aims to define the factors that affect students’ acceptance of a web-based learning management system and test the moderating effect of their academic involvement in the success of a WLMS. 477 valid questionnaires were collected from university/college students to empirically test the research model using the structural equation modelling approach. The results concludes that indirect and direct effects account for 49% of the variation in the intention to use, which is explained by technical system quality, information quality, educational quality, service quality of the technical support team and user satisfaction. High academic involvement moderates the impact of different service qualities of the WLMS on user satisfaction, intention to use the system, and success of the WLMS. Based on the findings, theoretical and managerial implications are discussed.

## 1 Introduction

Advances in technology and the increasing use of the Internet have impacted all aspects of our lives and education is no exception. Traditional classrooms are no longer constrained to conventional teaching methods and learning progress (Alagarsamy and Vijay, 2019; Alsayyari et al., 2019; Dobre, 2015). Technology mandated educational institutions to change their methods of teaching (Baragash and Al-Samarraie, 2018; Ching-Ter et al., 2017; Ramírez-Correa et al., 2017). The acronym ICT is taken to stand for information and communication technology, and it is defined as "technologies that are used for accessing, gathering, manipulating and presenting or communicating information. The technologies could include hardware (e.g. computers and other devices); software applications; and connectivity (e.g. access to the Internet, local networking infrastructure, videoconferencing)" (Lloyd, 2005). In accordance, several institutions integrated Information Communication Technology (ICT) to the physical classroom to strengthen teaching and streamline communication between the instructors and the students. However, further advances in technology ushered in a new set of challenges in implementing ICT (Granić and Marangunić, 2019; Hassanzadeh et al., 2012; Martins et al., 2019). To overcome these, the next step was to implement web-based classrooms to benefit from both instructor-led teaching and web resources (Al-Fraihat et al., 2020; Bouilheres et al., 2020; Naveed et al., 2020). This integration then necessitated the development of a web-based learning management system (WLMS) that could deliver instructor-led lessons using both the physical and online environment. WLMS also included tools for communication, data analytics, monitoring, feedback and reporting. A WLMS is an enhanced form of the e-learning concept (Granić and Marangunić, 2019; Greenhow et al., 2019; Martin and Bolliger, 2018) and uses various digital formats and communication tools to deliver lessons in a blended environment, that is, a combination of online and physical classrooms. Given that a majority of the top-ranked educational institutions had already implemented online-based teaching techniques, the next logical step was to invest in a WLMS to create and deliver teaching modules, evaluate the course progression, and provide real-time monitoring and feedback. With a WLMS, students can access a complete range of online resources to supplement classroom material, which in turn, leads to improved performance (Kasemsap, 2021; Stone and Zheng, 2014; Tsai et al., 2019).

Improving the process of teaching and learning is the goal of the instructors and technology is seen as a prominent component to make this a reality. In particular, the advent and development of ICT have made teaching activities more technology-based. However, the use of different delivery methods based on ICT “should not define the pedagogical practice.” Rather, technology should provide “the mechanism through which the teacher implements the best pedagogy for that course or topic”. The author argues that pedagogy (traditional learning, e-learning, and blended learning) should be explicitly considered to enhance learning (O'Neil and Fisher, 2008; Roddy et al., 2017). The instructor must use technology to strengthen the quality of the course. The instructor will also achieve a higher learning experience in different pedagogies by using technology.

A WLMS is “an information system that facilitates e-learning by supporting teaching and learning activities and the administration and communication associated with them” (Klobas and McGill, 2010). It is a structured internet-based educational program that regulates syllabus and supervises learning tasks (Stone and Zheng, 2014). Moreover, the WLMS combines functions such as study methods, course design, content management, student portal, and well-organized administration system (Al-Fraihat et al., 2020; Arenas-Gaitán et al., 2018; Greenhow et al., 2019). Besides, it also links students to the learning material in a structured way through specially designed web-based student learning software and programs (Arenas-Gaitán et al., 2018; Chaw and Tang, 2018; Isaac et al., 2019). These systems monitor learning activities and student performance by tracking system activities and showing statistics and strategies (Al-Fraihat et al., 2020; Kite et al., 2020; Marcano et al., 2019).

The emergence of scientific innovations has meant that everyone is granted fair opportunity to education regardless of the cultural or financial divisions. Undoubtedly, it will be less than desirable to utilize or incorporate these new technologies in education without evaluating the necessary conditions of pedagogy. The system may, in these situations, be used only as a promotional and not as an academic method (Cidral et al., 2018; Dorobat et al., 2019; Isaac et al., 2019). Most WLMSs are available in a variety of versions. Hence, investigating the key factors that influence WLMS performance is essential. Much of the literature reflects mainly on potential utility, projected convenience of usage, the mentality of consumers toward innovation and psychological motive (Al-Sharhan et al., 2020; Granić and Marangunić, 2019; Greenhow et al., 2019). This research work proposes a conceptual model and proves its possibility in the South Indian education systems.

The objectives of this study are: (a) to define the factors that influence the success of WLMS use in India's educational context; (b) to build and analyze a model to measure success of WLMS, integrating student expectation/actions and performance of the software system; (c) and to test the moderating effect of students' academic involvement in the success of a WLMS.

The current study is organized as follows. The first and second sections discuss the introduction and research aim, respectively. The third section provides the literature review, specifically the factors affecting WLMS success followed by the conceptual models. The next section explains the research method while the fifth provides detailed data analysis. The sixth section includes discussions, and the last discusses the theoretical and practical implications of the study. Finally, study concludes with limitations and directions for future research.

## 2 Literature review

The existing theoretical background has been adapted from various studies focused on the perspective of the information system. Similar approaches have been used to study e-learning transition, constant usage, and e-learning performance. Some of these include the Information System success model (DeLone and McLean, 2003), e-learning success model (Holsapple and Lee-Post, 2006), online communication success model (Lin and Lee, 2006), measuring online learning system success model (Lin, 2007), and measuring e-learning systems success models (Hassanzadeh et al., 2012).

### 2.1 Effectiveness of web-based learning systems

E-learning has become mainstream in the education sector, especially, finding favor in higher education. An instructor can use various technologies as means to impart e-learning. e-earning usually applies to all online learning that takes place via the internet. In reality, the e-learning system is a World Wide Web-based educational program which offers versatile student education (Al-Fraihat et al., 2020; Al-Sharhan et al., 2020; Kite et al., 2020).

The development of new technology has undoubtedly opened gates to provide anytime and anywhere learning, thus, extending reach to a widely dispersed population with access to the Internet, and a device such as laptop, computer, tablet or mobile. This being said, without understanding the critical features, the implementation of the most innovative and recent technologies is pointless. Rather than having any academic reach, such implementation may just be a promotional exercise. There is no question that in a dynamic and decentralized world, the Internet and other emerging technologies are enabling e-learning. Because of the variations in certain areas between conventional learning and e-learning, there is a need for efficient and productive transfer of conventional programs to e-learning. The shift may involve a complicated undertaking that involves adequate preparation, tracking, and supervision. Besides, the consistently increasing worldwide demand for e-learning has prompted the adoption of web-based environments; however, these need to be first evaluated based on their performance. The success of e-learning education depends highly on WLMS implementation and its adaptation by the end-users (Al-Fraihat et al., 2020; Kite et al., 2020; Naveed et al., 2020).

### 2.2 Theoretical foundation

Technology Acceptance Model and Unified Theory of Acceptance and Use of Technology models are the most popular theory-adopted models for technology acceptance-based research (Al-Fraihat et al., 2020; Šumak et al., 2011; Venkatesh and Davis, 2000). Although user acceptance and use are essential to quantify success, they are not the same as success (Petter et al., 2008; Sukendro et al., 2020). These models have been criticized by many researchers for poor fit, limited explanatory and predictive power, and lack of practical value (Abdullah et al., 2016; Al-Fraihat et al., 2020; Venkatesh and Davis, 2000).

User satisfaction approach is another significant direction of information system research (Abasi et al., 2015; Al-Fraihat et al., 2020). Satisfaction has been found to be a fundamental measure in the success, effectiveness, usage, and acceptance of information systems; however, it was not measured in the TAM and UTAUT models. Net benefit is regarded as one of the most critical measures of IS success, and it constitutes the extent to which an IS contributes to the success of various stakeholders, whether positive or negative. It has been measured by sometimes assessing the individual or organizational impact (Abasi et al., 2015; Petter et al., 2008). In WLMS, success is posited to influence both user satisfaction and their intentions to use the system. TAM and UTAUT models have limitations in measuring user satisfaction and net benefits, and for these reasons, many researchers have used the DeLone and McLean information systems success models to investigate the success of WLMS use in India's educational context.

One of the most commonly discussed and proven models in the Information System (IS) field is the DeLone and McLean model of information systems success (D&M IS) developed by DeLone & McLean, (1992). D&M IS model provides a detailed view of the performance of the information process. The original model contains six different elements of effective information systems: “System Quality, Information Quality, Use, User Satisfaction, Individual Impact, and Organizational Impact.” Revised D&M IS success model (2003) was introduced again with a new feature, service quality (DeLone and McLean, 2003). Throughout time, the authors have updated the success model of D&M IS to match the criteria provided by the multiple IS features as also the multiple perspectives that came to light between 2010 and 2020. The original model by DeLone and McLean (1992) used the corporate performance as a result of IS progress, and an upgrade, the DeLone and McLean (2003) model was revised to include the overall benefits to suit all IS environments.

### 2.3 Designing the conceptual model

We identified critical dimensions of IS success (information quality, system quality, education quality, service quality, system use/usage intentions, user satisfaction, and net system benefits) and the relationships among IS critical dimensions of success. The definitions of IS success dimensions, indicators, and the sources, were presented in Table 1.

**Table 1:** Components of research model.

Meaning	Indicators	Authors
**Technical System Quality**		
Accuracy of the technical system is process output in terms of efficiency, convenience to use, and other process metrics like flexibility, usability, user friendly, interactivity, system speed, and security. The technical quality of the system practically tests a technological success.	LMS availability, ease of use, user-friendliness, high-speed access to information, attractive features, reliability, and security	(Arenas-Gaitán et al., 2018; Costa et al., 2020; Hassanzadeh et al., 2012; Ramírez-Correa et al., 2017)
**Educational System Quality**		
The objective of quality of the educational system measures system quality based on the functionalities which encourage and enhance pedagogy.	Appropriateness to the context/pedagogies, effective collaboration, effective interaction between users, evaluation of learning performance and personalized information presentation	(Cidral et al., 2018; Costa et al., 2020; Hassanzadeh et al., 2012; Wang and Liao, 2008)
**Content and Information Quality**		
The quality of content and information is the output of the model, and it tests conceptual efficiency.	Usefulness, updated information, accuracy and precise information, better display, useful format, and organized content	(Efiloğlu Kurt, 2019; Holsapple and Lee-Post, 2006; Ramírez-Correa et al., 2017; Wixom and Todd, 2005)
**Service Quality**		
It is user assistance using the education program, and support to use the program, which is an essential service for program users. While some scholars believe that service quality is a part of model system quality, but in the past few years, it has been an individual variable of the growing nature of information systems.	Better support by staffs on explanation, staff availability, interaction by the team on LMS development, suggestion on future enhancement.	(Klobas and McGill, 2010; Lin and Lee, 2006; Wang and Wang, 2009)
**User Satisfaction**		
User satisfaction is the general perception of consumers regarding the process, which is also used to assess the students' mindset. Satisfaction component evaluates interaction between user and WLMS. User satisfaction is regarded as among the five essential foundations of efficiency in web-based education.	The frequency of use, dependency, voluntary, mandatory, and intent to use	(Costa et al., 2020; DeLone and McLean, 2016; Hassanzadeh et al., 2012; Ho and Dzeng, 2010)
**Intention to Use**		
Intention to use is the choice to implement a device when the individual finally uses something, and it is expected that it would be in the long run. Intention to use is a mentality.	System efficiency, user confidence, user needs, positive attitude, perceived utility, user satisfaction, recommending others	(Chen et al., 2019; Holsapple and Lee-Post, 2006; Sukendro et al., 2020)
**Net Benefits**		
Using the WLMS program, net profits are from the effect of an e-learning system on a single individual, company, entity, business, or society. Through the flow of time, the advantages of using the system go out of an individual's control and gradually extend to organizations and societies.	Improved learning performance, problem-solving, quick response, enhancement in the competitiveness of the college	(DeLone and McLean, 2016; Lin, 2007; Petter et al., 2008; Sukendro et al., 2020)
**Academic Involvement**		
The involvement is defined as the ability of students to engage in their daily academic activities, such as attending lectures, submitting assignments, and following teacher instruction in class. It is used as a measure of the quality of institutional teaching.	Measures includes affective and cognitive relevance based on inherent needs, values, and interest.	(Avcı and Ergün, 2019; Baragash and Al-Samarraie, 2018; McQuarrie and Munson, 1992; Venugopal and Jain, 2016)

#### 2.3.1 Relationship between critical dimensions of IS success

Holsapple & Lee-Post (2006) updated the success model of D&M IS to assess e-learning courses (Holsapple and Lee-Post, 2006). Holsapple and Lee-Post's (2006) e-learning success model covers factors such as, technical efficiency, knowledge efficiency, support consistency, customer retention, utilization and gains or losses; however, it neglects to address the e-learning systems' instructional material (Holsapple and Lee-Post, 2006). No reason is provided for the relationship that exists between the dimensions. Eventually, Lin & Lee (2006) proposed an updated approach to investigate the factors influencing the effective usage of online group, utilizing the success model for D&M IS (Lin and Lee, 2006). Their concept of effective online communication incorporates identical elements to the updated D&M IS success model. D&M IS model, however, has neglected to address the aspect of quality of education and net profit. Apart from these improvements, it is nearly similar to the successful model of the updated D&M IS. Each of these models is based on both old and revised DeLone and IS success models from McLean (1992, 2003). Furthermore, both the old and revised D&M IS success models were built to test the overall progress of the IS program, not directly to examine the WLMS or other e-learning programs. Therefore, the standard of education was not addressed in either of these models (Abasi et al., 2015; DeLone and McLean, 2003).

Lin & Lee (2006) and Wang & Liao (2008) showed that user loyalty can be assessed using metrics such as user attachment and user engagement, and input into an information network (Lin and Lee, 2006; Wang and Liao, 2008). From the other side, under the purpose of using element (device utilization), Wang et al. (2007) analyzed device dependence, and user participation in IS, which was evaluated within consumer satisfaction by evaluating expected usefulness (Wang et al., 2007). Additionally, consumer engagement was calculated in the context of the users' willingness to enthusiastically use the system. Hassanzadeh et al. (2012) found that target accomplishment reflects professional and educational success objectives. Within the same report, the standard of education was also evaluated by learning assessment, program efficiency, joint research, and constructive learning, which is close to achieving the educational target. From the above discussions, it is evident that the updated D&M IS success model would be a better fit to assess WLMS progress if the educational quality aspect was included. According to Hassanzadeh et al. (2012), when calculating effective e-learning programs, an additional element such as user engagement or target accomplishments is viewed as an intrinsic part of educational quality, user fulfillment, and motivation to use components (Hassanzadeh et al., 2012). Adding more variables into the current updated D&M IS success model will impact the most critical objective of the research model by misleading the participants, which will adversely impact the model's credibility and efficiency (Alagarsamy and Vijay, 2019).

#### 2.3.2 Academic involvement and learning management system success

Many studies define student academic involvement as student engagement (Johnson et al., 2015; Kim and Frick, 2011; Venugopal and Jain, 2016). Additionally, Kuh & Hu (2001) state that academic involvement is the degree to which students connect with their educational activities, and that participation is positively related to a variety of desirable outcomes, including high grades, student satisfaction, and perseverance. Various forms of technologies are used to enhance the teaching and learning experience (Martin and Bolliger, 2018; Meyer, 2014). Literature clears that interactive lectures lead to a high level of academic involvement (Manwaring et al., 2017; Venugopal and Jain, 2016). Many studies conclude that social media can be an essential educational tool to improve academic involvement and communication between students and teachers (Greenhow et al., 2019; Selwyn and Stirling, 2016; Tess, 2013).

Research indicates that students showed a positive attitude towards the use of mobile devices in the classroom (Al-Emran et al., 2016; Ching-Ter et al., 2017; Granić and Marangunić, 2019). Mobile devices are being increasingly used to generate interest among students (Al-Emran et al., 2016; Sung et al., 2016). To support the change, several higher educational institutions are now investing in a WLMS. WLMS has the potential to improve the way students work together, connect with instructors, and access the resources they use to learn (Al-Sharhan et al., 2020; Granić and Marangunić, 2019; Heflin et al., 2017). It enhances the teaching and learning process profoundly by providing a large variety of opportunities. Like other types of technologies, it is believed that a WLMS will have a positive effect on student involvement (Granić and Marangunić, 2019; Kite et al., 2020; Malm and Defranco, 2012). It is, therefore, clear that academic involvement can be improved by WLMS, and high academic involvement can strengthen the WLMS success. Based on the above discussions, the below hypotheses were proposed.

H1 *Different types of IS qualities (Technical system quality, Information quality, Educational system quality and Service quality) positively influence user satisfaction*

H2 *Different types of IS qualities (Technical system quality, Information quality, Educational system quality and Service quality) positively influence intention to use the system*

H3 *User satisfaction positively influences intention to use the system*

H4 *User satisfaction positively influences net user benefit*

H5 *Intention to use the system positively influences net user benefit*

H6 *Students' academic involvement strengthens WLMS success in higher education*

**Figure 1:**
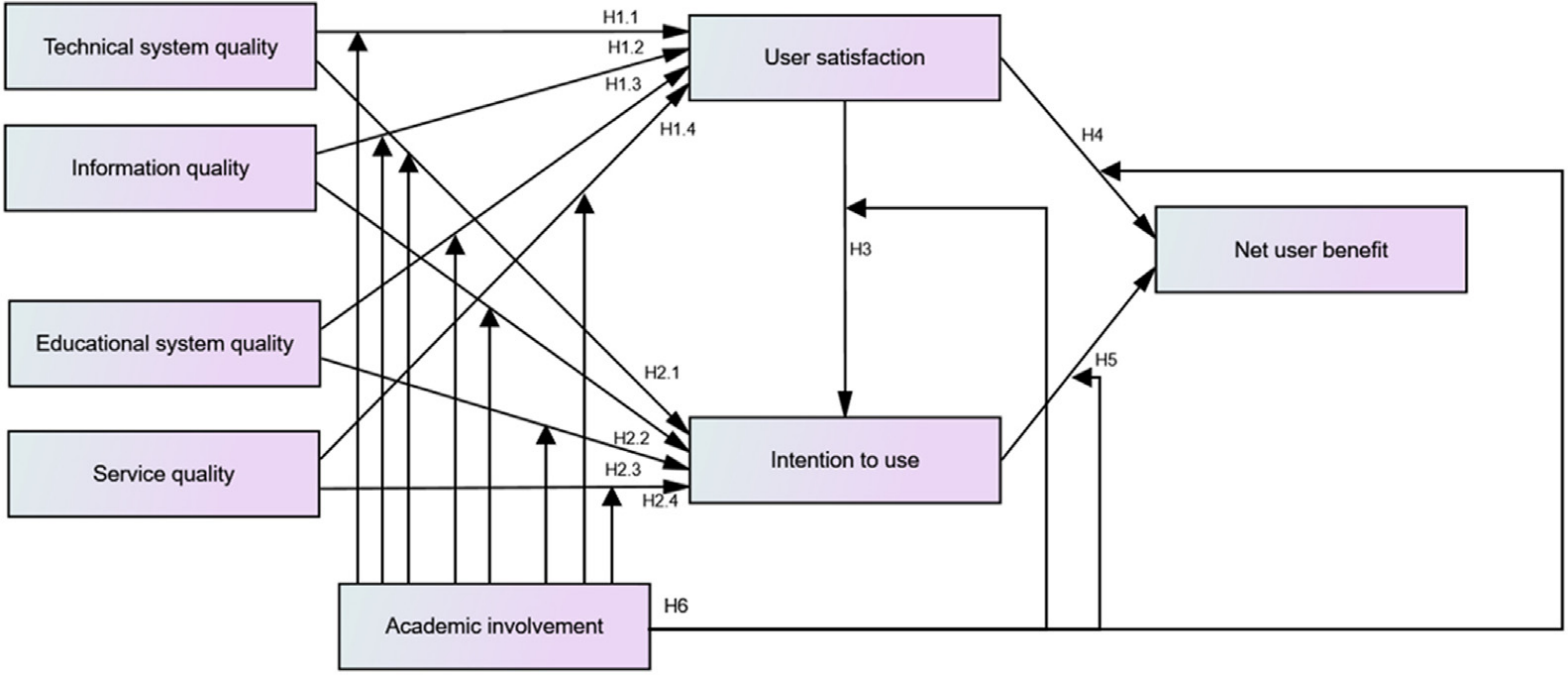
Conceptual model.

Therefore, in current research work, we use principles and models described in the previous studies, taking into consideration the opinions of students; and include a basis for assessing the success of WLMS. Our findings add richness to the existing literature. Based on literature analysis, we devise the original conceptual model, as described in Figure 1. As the figure shows, the models include all parameters of the extensively used D&M IS model for calculating WLMS success. Additionally, a different relation between intention to use and user satisfaction is incorporated into the previous D&M IS success model.

## 3 Methods

### 3.1 Sample and data collection

The hypotheses and conceptual model were tested using the positivist approach. A quantitative methodology was adopted, and an online survey was designed to collect the responses from university/college students from Tamil Nadu, India. Many higher education institutions have started using WLMS to support education, and accordingly, Tamil Nadu is in the transition state from traditional educational practices to web-based teaching practices and this is why educational institutions in the state were considered as a study population. Samples were collected from students enrolled in the WLMS. Data was collected from September 2019 to March 2020. A total of 512 samples were collected. As part of the data clearing process, 44 samples were removed due to missing values and multivariate outliers. For the final data analysis, only 477 responses were used. The respondents belonged to under-graduate and post-graduate arts, science, and engineering programs. The rate of useful response was thus 93%.

### 3.2 Measures

A well-structured questionnaire was used to measure WLMS success, using an instrument adopted from multiple previous studies. In present study, adapted survey instruments were applied to measure the factors predicting the success of WLMS usage. Participants were asked to indicate on a seven-point scale (1-strongly disagree, 7-strongly agree) based on their level of agreement about the WLMS success components and academic involvement. The research constructs and indicators are presented in Table 2. Statistical analysis like cluster analysis and confirmatory factor analysis and structural equation modelling were used using IBM SPSS 25 and AMOS 25 software.

**Table 2:** Measures and indicators.

Constructs	Items	Source of adoption
Technical System Quality (6 items)	The WLMS availability is very high	Hassanzadeh et al. (2012); Wang and Wang (2009)
	The WLMS is easy to use	
	The WLMS is user-friendly	
	The WLMS provides high-speed access to information	
	The WLMS has attractive features to appeal to the users	
	The WLMS is reliable and secured	
Educational System Quality (5 items)	The WLMS is appropriate with students' learning styles	Hassanzadeh et al. (2012); Lee (2010); Wang & Liao (2008)
	The WLMS provides collaborative and active learning	
	The WLMS offers interactive features between users and the system	
	The WLMS delivers the possibility of evaluation of learning and performance	
	The WLMS provides a personalized information presentation	
Information Quality (7 items)	I think course content is always presented in a useful format	Hassanzadeh et al. (2012); Lin (2007); Wang et al. (2007); Wang and Liao (2008)
	The WLMS provides up-to-date information	
	The WLMS provides course accurate content/information	
	The WLMS provides precise content/information	
	The WLMS provides content you need at the right time	
	The WLMS provides information that is easy to understand	
	The WLMS provides organized content/information	
	The course content is presented in a useful format in the WLMS	
Service Quality (5 items)	The IT department/e-learning support staff provides a proper level of assistance and explanation	Hassanzadeh et al. (2012); Lin (2007); Wang and Wang (2009)
	The IT department/e-learning support staff are always available for consultation	
	The IT department/e-learning support staff provide satisfactory support to users using the WLMS at College/University	
	The WLMS developers interact extensively with users during the development of the e-learning system	
	The IT department/e-learning support staff responds cooperatively to your suggestion for future enhancements of the WLMS	
Intention to use (6 items)	The frequency of using the WLMS is high	DeLone and McLean (2016); Hassanzadeh et al. (2012); Ho and Dzeng (2010); Holsapple and Lee-Post (2006)
	If the WLMS was not mandatory, I would still use it	
	I spend many hours per week with the WLMS	
	Assuming I have access to the WLMS, I intend to use it	
	The WLMS usage is voluntary	
	I depend on the WLMS	
User satisfaction (6 items)	The WLMS is efficient	Hassanzadeh et al. (2012); Holsapple and Lee-Post (2006); Lee (2010)
	The WLMS helps to gain my confidence	
	The system is adequate to meet the educational needs of the users	
	Most of the users bring a positive attitude or towards the WLMS function	
	I think that the perceived utility about the WLMS is high	
	Overall, I am satisfied with the system performance	
Net Benefits (8 items)	The WLMS helps me improve my learning performance	Hassanzadeh et al. (2012); Lin (2007); Wang and Wang (2009)
	The WLMS helps me think through problems	
	The WLMS enables the College/University to respond more quickly to change regarding teaching and learning	
	The WLMS helps to enhance the competitiveness of the College/University	
	The WLMS allows the College/University to save cost relating to teaching and learning	
	The WLMS helps the College/University to speed up transactions or shorten product cycles (change the words)	
	The WLMS helps the College/University increase return relating to teaching and learning investment	
	The WLMS helps the College/University to achieve its goal	
Academic Involvement (8 items)	Interesting: Boring	Wells and DauntEduscape (2016); Zaichkowsky (1994)
	Relevant: Irrelevant	
	Exciting: Unexciting	
	Means a lot to me: Means nothing to me	
	Appealing: Unappealing	
	Fascinating: Mundane	
	Valuable: Worthless	
	Needed: Not needed	

## 4 Results

### 4.1 Validity and reliability analysis

Scholars suggest that adopted scales with sufficient empirical and theoretical evidence can be taken directly for confirmatory factor analysis without running exploratory factor analysis beforehand (Hurley et al., 1997). The maximum likelihood estimator was used to test the measurement model and structural model using AMOS 25; however, the present study violated the multivariate normality assumptions, with insufficient sample size to apply distribution-free estimation methods in IBM AMOS 25. Thus, to fix normality issue, the maximum likelihood estimation with the bootstrap resampling method of 2000 samples (most widely used sampling size in bootstrapping technique) was used to obtain an accurate estimation of standard errors, as reflected in the p values and confidence intervals (Arifin and Yusoff, 2016). The bias-corrected confidence interval was set at the 95% confidence level.

Confirmatory Factor Analysis (CFA) is mainly used to define the factor structure of data. Since the instrument was adopted from previous studies and the factor structures were specified in the instrument, exploratory factor analysis was not done, and CFA was used to confirm the defined factor structure (Brown, 2015). Study allows us to check the construct validity as well as the reliability of the research instrument. Convergent and discriminant are the subtypes of construct validity. Cronbach's Alpha coefficient (α) was used to estimate the internal reliability of the instrument (NUNNALLY, 1975). Construct validity indicates that a questionnaire intended to assess a distinct construct (i.e., educational quality) is estimating that construct. Convergent validity means variables are correlated adequately with each other within their parent constructs, and the latent factor is explained well by the observed variables. Conversely, discriminant validity of the underlying factor is adequately described by other variables than by its observed variables. Both validities are essential for optimum construct validity (Campbell and Fiske, 1959; Churchill, 1979). Composite Reliability (CR), Average Variance Extracted (AVE), and Maximum Shared Variance (MSV) are a few measures that are mainly used for confirming validity and reliability (Flury et al., 1988).

Table 3 shows that standardized loading estimates (β) was above 0.5 except for IQ6 (WLMS provides information that is easy to understand); however, all the items were significant at 1%, and so we retained all of them. The Cronbach alpha coefficient for all constructs was more than 0.7 (Cronbach α > 0.7). The properties of the measurement model were evaluated with Composite reliability (CR) and convergent validity (Hair et al., 2014), presented in Table 4. All constructs exhibited CR with the minimum acceptable level of 0.7 (CR > 0.7), indicating excellent composite reliability. The AVE measure was assessed for the estimation of scales' convergent validity (Fornell and Larcker, 1981). The latent construct's Average Variance Extracted (AVE) values must be greater than 0.5 (AVE >0.5) to explain on average at least half of the variance of indicators in the research (Hair et al., 2014). The AVE values (0.5) for all constructs were higher than normal levels, thus, supporting the convergent validity of the constructs (see Table 4).

**Table 3:** Confirmatory factor analysis loading and Initial Reliability.

Code	Constructs	β	Indicator Reliability	Cronbach Alpha	Mean (SD)
TSQ1	Technical System Quality	0.82	0.86	0.888	6.2 (0.4)
TSQ2		0.69	0.879		
TSQ3		0.88	0.851		
TSQ4		0.64	0.878		
TSQ5		0.69	0.879		
TSQ6		0.81	0.859		
ESQ1	Educational System Quality	0.82	0.885	0.907	6.4 (0.6)
ESQ2		0.75	0.896		
ESQ3		0.86	0.877		
ESQ4		0.8	0.886		
ESQ5		0.83	0.884		
IQ1	Information Quality	0.72	0.875	0.889	6.4 (0.4)
IQ2		0.58	0.882		
IQ3		0.77	0.871		
IQ4		0.6	0.88		
IQ5		0.87	0.862		
IQ6		0.44	0.893		
IQ7		0.78	0.87		
IQ8		0.85	0.865		
SQ1	Service Quality	0.78	0.839	0.871	6.4 (0.5)
SQ2		0.71	0.862		
SQ3		0.71	0.852		
SQ4		0.85	0.827		
SQ5		0.78	0.84		
ITU1	Intention to use	0.61	0.902	0.91	6.3 (0.5)
ITU2		0.84	0.888		
ITU3		0.87	0.882		
ITU4		0.88	0.882		
ITU5		0.96	0.872		
ITU6		0.63	0.903		
US1	User satisfaction	0.58	0.88	0.885	6.4 (0.4)
US2		0.63	0.873		
US3		0.7	0.86		
US4		0.68	0.861		
US5		0.9	0.858		
US6		0.89	0.86		
NB1	Net Benefits	0.86	0.92	0.933	6.4 (0.5)
NB2		0.77	0.924		
NB3		0.88	0.918		
NB4		0.87	0.92		
NB5		0.71	0.931		
NB6		0.84	0.92		
NB7		0.78	0.925		
NB8		0.7	0.931		
AINV1	Academic Involvement	0.76	0.877	0.893	5.3 (0.4)
AINV2		0.66	0.884		
AINV3		0.84	0.869		
AINV4		0.7	0.881		
AINV5		0.66	0.886		
AINV6		0.67	0.883		
AINV7		0.74	0.878		
AINV8		0.72	0.881

**Table 4:** Reliability and validity measures.

Constructs	CR	AVE	MSV	1	2	3	4	5	6	7	8
1. Net Benefits	0.936	0.647	0.426	**0.804**							
2. Academic Involvement	0.895	0.517	0.462	0.537∗∗	**0.719**						
3. Information Quality	0.890	0.512	0.357	0.439∗∗	0.545∗∗	**0.716**					
4. Technical System Quality	0.890	0.577	0.155	0.277∗∗	0.327∗∗	0.356∗∗	**0.759**				
5. Educational System Quality	0.907	0.662	0.187	0.170∗∗	0.471∗∗	0.205∗∗	0.248∗∗	**0.814**			
6. Intention to Use	0.917	0.654	0.425	0.460∗∗	0.679∗∗	0.532∗∗	0.393∗∗	0.432∗∗	**0.809**		
7. User Satisfaction	0.877	0.549	0.426	0.653∗∗	0.607∗∗	0.494∗∗	0.320∗∗	0.389∗∗	0.506∗∗	**0.741**	
8. Service Quality	0.876	0.587	0.425	0.442∗∗	0.621∗∗	0.597∗∗	0.281∗∗	0.382∗∗	0.652∗∗	0.580∗∗	**0.766**

Note: ∗p < 0.05; ∗∗p < 0.01; Diagonal value represent the square root of AVE.

Diagonal value represent the square root of AVE and represented in **BOLD**.

Table 4 shows that Maximum Shared Variance (MSV) is less than AVE; the square root of AVE is greater than the inter-construct correlations, thus, supporting the discriminant validity of the constructs (Hair et al., 2014). These results support the validity of the constructs and reliability of the instrument. χ^2^/d.f (2 < χ^2^/df ≤ 3), Normed Fit Index (.90 ≤ NFI <.95) Comparative Fit Index (.95 ≤ CFI <.97), Goodness-of-Fit Index (.90 ≤ GFI <.95), Standardised Root Mean square Residual (.05 < SRMR ≤.10), and Root Mean Square Error of Approximation (.05 < RMSEA ≤.08) are used as indicative of good fit (Hu and Bentler, 1999). These indexes are the most commonly published model fit indices. χ^2^ (2728.189)/d.f (1246) = 2.19; CFI = .912; NFI = .907; GFI = .901; SRMR = .016; and RMSEA = .050; these results indicate that the measurement model is a good fit.

### 4.2 Structural model

**Figure 2:**
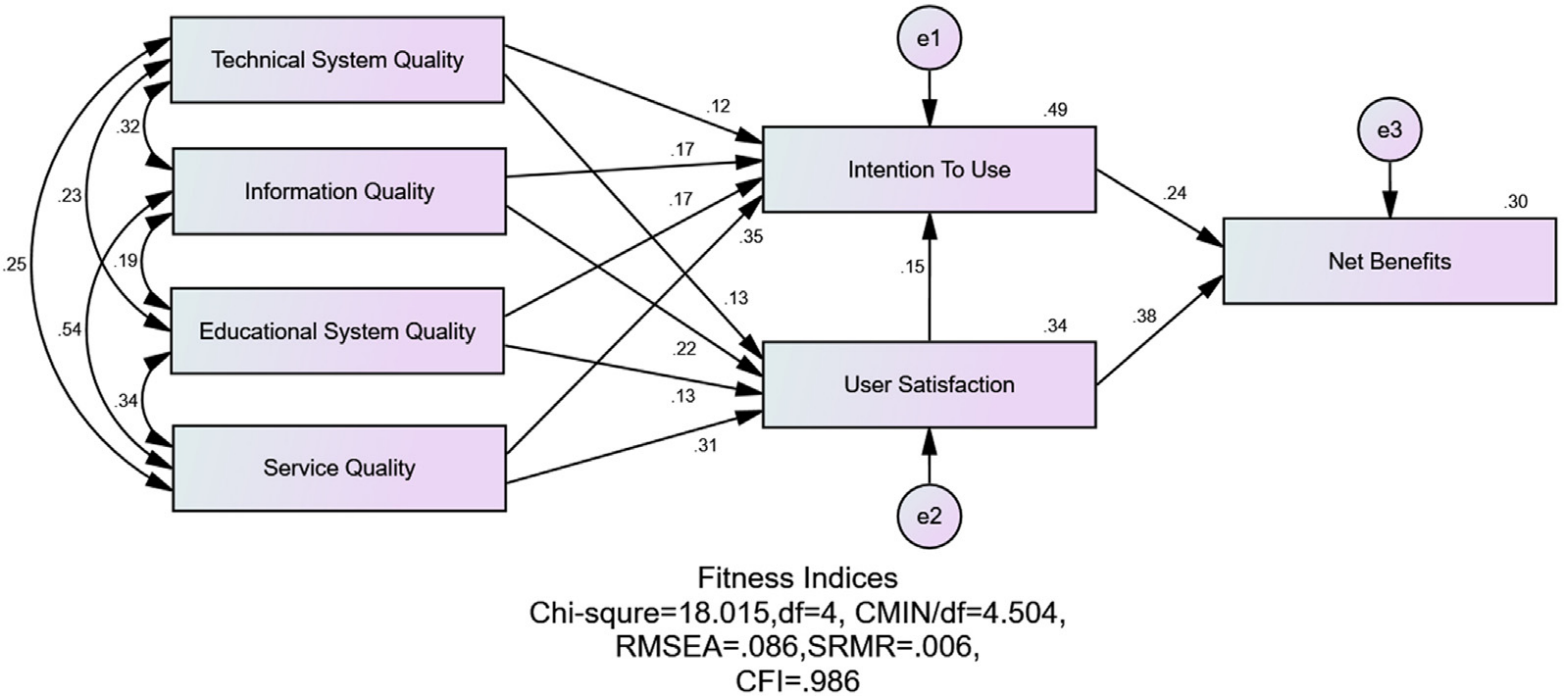
Hypothesized model.

The study hypotheses were tested using structural modelling. Before testing the hypothesis, the model goodness of fit was assessed using the same model fit indices used in the above measurement model. The model possessed adequate goodness of fit with values for, GFI = .990, NFI = .982, CFI = .986, RMSEA = .086, SRMR = .006 and CMIN (18.015)/df (4) = 4.47. The standardized path coefficient (β), error of prediction (e1, e2, e3) and the coefficient of determinant (R^2^) are presented in Figure 2 and Table 5. The coefficient of determinant is the proportion of variation in the response variable explained by the model, and the standardized path coefficient compares the strength of the effect of each independent variable to the dependent variable.

**Table 5:** Hypothesis testing.

Paths	Direct Effect	Indirect Effect	R^2^	Result
Technical System Quality → User Satisfaction	0.126∗∗	-	0.341	Supported
Information Quality → User Satisfaction	0.224∗∗	-		Supported
Educational System Quality → User Satisfaction	0.133∗∗	-		Supported
Service Quality → User Satisfaction	0.311∗∗	-		Supported
Technical System Quality → Intention to Use	0.123∗∗	0.019∗∗	0.49	Supported
Information Quality → Intention to Use	0.172∗∗	0.033∗∗		Supported
Educational System Quality → Intention to Use	0.175∗∗	0.02∗∗		Supported
Service Quality → Intention to Use	0.354∗∗	0.046∗∗		Supported
User Satisfaction → Intention to Use	0.148∗∗	-		Supported
User Satisfaction → Net Benefits	0.381∗∗	0.036∗∗	0.297	Supported
Intention to Use → Net Benefits	0.243∗∗	-		Supported
Technical System Quality → Net Benefits	-	0.082∗∗		Supported
Information Quality → Net Benefits	-	0.135∗∗		Supported
Educational System Quality → Net Benefits	-	0.098∗∗		Supported
Service Quality → Net Benefits	-	0.216∗∗		Supported

∗∗p < 0.01.

The students' user satisfaction towards the WLMS is significantly and positively influenced by the technical system quality (β = 0.126; p < 0.01), information quality (β = 0.224; p < 0.01), educational quality (β = 0.133; p < 0.01) and service quality of the technical support team (β = 0.311; p < 0.01). Thirty-four percent of the variation in user satisfaction is explained by technical system quality, information quality, educational quality and service quality of the technical support team; however, the service quality of the technical support team and information quality are the most significant predictors of user satisfaction with the highest standardized path coefficient. These results support H_1,_ refer to Table 4. The students’ intention to use the WLMS for future purpose is significantly and positively influenced by the technical system quality (β = 0.123; p < 0.01), information quality (β = 0.172; p < 0.01), educational quality (β = 0.175; p < 0.01) and service quality of the technical support team (β = 0.354; p < 0.01). Apart from those four constructs, user satisfaction is also seen to significantly and positively influence the intention to use dimension (β = 0.148; p < 0.01). Technical system quality (β = 0.019; p < 0.01), information quality (β = 0.033; p < 0.01), educational quality (β = 0.02; p < 0.01), and service quality of the technical support team (β = 0.046; p < 0.01) have a significant and indirect effect on the intention to use dimension through user satisfaction.

Altogether, indirect and direct effects account for 49% of the variation in the intention to use, which is explained by technical system quality, information quality, educational quality, service quality of the technical support team and user satisfaction; however, the service quality of the technical support team contributes more to predictors of intention to use than other predictors with the highest standardized path coefficient and supports H_2_, refer to Table 5.

Hypotheses H_4_ and H_5_ are also supported, showing that the net benefit of WLMS is significantly and positively influenced by the user satisfaction about WLMS (β = 0.381; p < 0.01) and the intention to use (β = 0.243; p < 0.01). Note that user satisfaction contributes more to net benefit than the intention to use. However, the net benefit is indirectly influenced by technical system quality (β = 0.082; p < 0.01), information quality (β = 0.135; p < 0.01), educational quality (β = 0.098; p < 0.01), service quality of the technical support team (β = 0.216; p < 0.01) through user satisfaction and intention to use. User satisfaction has an indirect effect on net benefit through intention to use (β = 0.036; p < 0.01). Altogether, indirect and direct effects account for 30% of the variation in the net benefits.

### 4.3 Moderation effect of academic involvement on WLMS success

**Figure 3:**
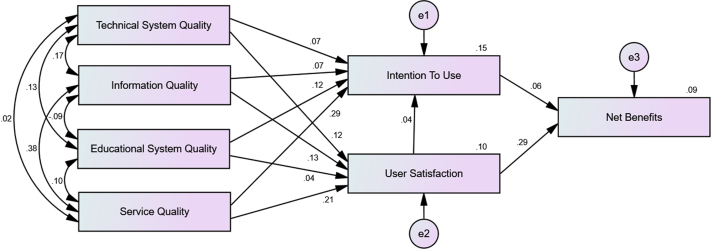
Hypothesized model for low academic involvement.

**Figure 4:**
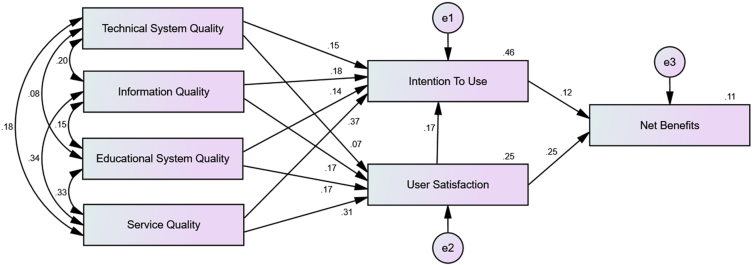
Hypothesized model for high academic involvement.

Cluster analysis collectively looks at the profiles of each observed respondent and group as a simpler response. Zaichkowsky's Personal Involvement Inventory measures were used for cluster analysis. WLMS can improve the way students work together, connect with instructors, and access the resources they use to learn. Previous studies conclude that students' academic involvement and WLMS success were related; WLMS success might differ based on students' academic involvement level (Al-Sharhan et al., 2020; Granić and Marangunić, 2019; Heflin et al., 2017). Students' academic involvement was classified into categorical variables using cluster analysis. The cluster analysis showed the presence of the two clusters. The quality of the solution was fair and based on Gentle, Kaufman, Rousseuw (1991) regarding the interpretation of cluster structures. Students in cluster 1 had a low level of academic involvement (n = 305), and those in cluster 2 had a high level of academic involvement (n = 172). These two clusters were used in the multi-group analysis to verify the moderation effect. The multi-group invariance analysis was conducted to determine the difference between the high and very high personal academic involvement samples and the link between the WLMS success model using Amos graphics. The p-value of the chi-square difference test [Unconstrained model (χ^2^ = 21.27, d.f = 8) and constrained model (χ^2^ = 48.685, d.f = 19)] is significant (p = 0.004), meaning the model differs across the group. The detailed results are presented in Table 6 and Figures 3 and 4.

**Table 6:** Multigroup analysis between low and high academic involvement group samples.

Paths	Low Involvement β_1_ (n = 305)	R^2^	High Involvement β_2_ (n = 172)	R^2^	Difference in Beta	ΔR^2^	P-Value for Difference
Technical System Quality → User Satisfaction	0.118∗	0.104	0.072	0.251	0.046	0.147∗∗	0.412
Information Quality → User Satisfaction	0.129∗		0.168∗		-0.039		0.735
Educational System Quality → User Satisfaction	0.036		0.170∗		-0.134		0.074
Service Quality → User Satisfaction	0.208∗∗		0.309∗∗		-0.101		0.397
Technical System Quality → Intention to Use	0.073	0.149	0.149∗	0.455	-0.076	0.306∗∗	0.349
Information Quality → Intention to Use	0.073		0.183∗∗		-0.110		0.118
Educational System Quality → Intention to Use	0.125∗		0.140∗		-0.015		0.299
Service Quality → Intention to Use	0.292∗∗		0.367∗∗		-0.075		0.152
User Satisfaction → Intention to Use	0.039		0.169∗∗		-0.130		0.078
Intention to Use → Net Benefits	0.060	0.093	0.123	0.109	-0.063	0.016	0.629
User Satisfaction → Net Benefits	0.290∗∗		0.255∗		0.035		0.850

Note: ∗p < 0.05; ∗∗p < 0.01 [∗, ∗∗ denotes the significant impact of the independent variable on the dependent variable at 5% and 1% significance level respectively].

The impact of technical service quality on user satisfaction is significant for the low academic involvement group (β_1_ = 0.118; p < 0.05) but not for the high academic involvement group (β_2_ = 0.072; p = 0.301). Information quality (β_1_ = 0.129; p < 0.05; β_2_ = 0.168; p < 0.05) and service quality of the technical support (β_1_ = 0.208; p < 0.01; β_2_ = 0.309; p < 0.01) are seen to be positively influencing user satisfaction for both academic involvement groups. The positive impact of educational service quality on user satisfaction is stronger for high academic involvement group (β_2_ = 0.170; p < 0.05), but not so for the low academic involvement group (β_1_ = 0.036; p = 0.582). The high academic involvement group has better prediction ability than a low academic involvement group on the relationship between educational service quality and user satisfaction. The low academic involvement group has better prediction ability than a high academic involvement group on the relationship between technical service quality and user satisfaction.

The positive impact of technical service quality (β_2_ = 0.149; p < 0.05), information quality (β_2_ = 0.183; p < 0.01) and user satisfaction (β_2_ = 0.169; p < 0.01) on the intention to use is stronger for high academic involvement group, hence, academic involvement moderates the impact of technical service quality, information quality and user satisfaction on WLMS intention to use. Educational service quality (β_1_ = 0.125; p < 0.05; β_2_ = 0.140; p < 0.05) and service quality of the technical support (β_1_ = 0.292; p < 0.01; β_2_ = 0.367; p < 0.01) are seen to be positively influencing the intention to use factor for both academic involvement groups; hence, academic involvement is not moderating the impact of educational service quality and service quality of the technical support on WLMS intention to use.

The WLMS intention to use doesn't have a significant impact on net benefits (β_1_ = 0.06; p = 0.244; β_2_ = 0.123; p = 0.186) for both academic involvement groups. Also, the WLMS user satisfaction positively influences the net benefits (β_1_ = 0.290; p < 0.01; β_2_ = 0.255; p < 0.01) for both academic involvement groups. These results show that academic involvement does not moderate the impact of user satisfaction and intention to use on net benefits. Hence, H_12_ is supported.

## 5 Discussions

In India, the e-learning industry is a prolific one, showing a steady growth rate of 25 percent year-on-year (Kandhari, 2020). The education system relied on conventional classroom-based learning until the end of the last decade; however, the Internet and the rise of digital technology have brought about a tactic change with several educational institutes gradually moving from conventional learning methods to digital learning. This change was in step with the evolving times and necessary to impart global skills to the students. The traditional classroom that was once characterized by dull, hour-long sessions was rejuvenated with the use of digital media. Digital education has made life easier for both students and teachers. However, the use of digital technology is still at a nascent stage in many educational institutions in southern parts of the country in states such as Tamil Nadu. To increase its use, the present study defines the factors that affect student acceptance of WLMS in India's educational context. Hypothesis testing results show that technical service quality, informational service quality, educational service quality and the service quality offered by the technical staffs to help the end-users gain familiarity with the WLMS are essential factors that positively influence student acceptance of WLMS. In particular, educational service quality and the service quality offered by the technical staff to help end-users are the essential factors that affect user satisfaction and intention to use. Since the e-learning concept is relatively new, students need support to accept its use. Also, WLMS provides interactive features to support communication between students, instructors, and as well as external and internal communities. Students gain greater control over their learnings, thus, ensuring its greater acceptance.

Based on the structural model, it is clear that technical service quality, informational service quality, educational service quality and the service quality offered by the technical staffs are the necessary components of measuring the success of WLMS and have a direct effect on user satisfaction and intention to use the system. The success of the WLMS is measured using net benefits received by the end-users; net benefit is directly and indirectly influenced by the technical service quality, informational service quality, educational service quality, and the service quality offered by the technical staff. Finding shows that the availability of reliable and secured user-friendly WLMS influences end-user satisfaction and future intentions to use the system. Accurate and up to date course contents also influence students' satisfaction towards a WLMS. As discussed in the above section, the different teaching facilities embedded in a WLMS and the associated technical support have a direct effect on user satisfaction, which in turn, leads to the intention to use the system. User satisfaction is a primary determinant of student willingness to use the system and achieve net benefits. Students are more likely to accept the WLMS when they feel that it simplifies and supports their learning journeys while making them more relevant, contextual, immersive and exciting.

Net benefit refers to the ability of the students to achieve their educational and personal goals. The model shows that user satisfaction directly and indirectly positively influences the net benefits achieved by the students. Hence, it explains user acceptance, integrating student actions and performance of the software system, and the second objective is achieved. These results are consistent with existing literature in the context of developing countries (Abasi et al., 2015; Arenas-Gaitán et al., 2018; DeLone & McLean, 2003, 2016; Hassanzadeh et al., 2012; Holsapple and Lee-Post, 2006; Lin and Lee, 2006; Ramírez-Correa et al., 2017; Wang and Wang, 2009; Wang et al., 2007; Wang and Liao, 2008). Literature indicates that different system qualities directly impact the intention to use when users start learning and using the system during the initial stages (Al-Sharhan et al., 2020; Arenas-Gaitán et al., 2018; Kite et al., 2020; Ramírez-Correa et al., 2017; Wang and Wang, 2009; Wang et al., 2007).

Nonetheless, the direct effect becomes less prevalent and is eventually replaced by an indirect effect through user satisfaction. As users gain knowledge with a new system, their instrumental concerns outweigh the system's concerns about the efficiency of use. Thus, students are more likely to continue using the system if they find it useful. The WLMS also offers other benefits, such as reduced costs for instance, because it cuts down on the need to use paper. Besides, students can access a sea of reference material with a few clicks of a mouse. Correspondingly, the higher the WLMS use, the more the advantages gained.

The third objective explains the moderating effects of academic involvement in the WLMS success model. For this purpose, multi-group analysis is performed. The result shows that students' academic involvement moderates the impact of technical and educational service quality on user satisfaction as also the impact of technical, information service quality and user satisfaction on intention to use. The positive impact of educational service quality on user satisfaction is stronger for the high academic involvement group. The positive impact of technology and information service quality on the intention to use is also stronger for the high academic involvement group. Finally, the positive relationship between user satisfaction and intention to use the system is stronger for the high academic involvement group. These results are almost consistent with the previous studies (Granić and Marangunić, 2019; Klobas and McGill, 2010; Malm and Defranco, 2012).

Academic involvement is an indicator that combined academic identification and academic participation can strengthen the relationship between the independent and dependent variables. In the present study, it is clear that user satisfaction, intention to use the system, and success of the WLMS are positively influenced by the different service qualities of the WLMS. When students work effort both inside and outside of school, including hours spent on homework, meeting deadlines, not skipping classes, getting along with teachers, having an interest in the subject matter, and related behaviours and attitudes are higher, there is higher academic involvement. The high academic involvement group strengthens the impact of different service qualities of the WLMS on user satisfaction, intention to use the system, and success of the WLMS both directly and indirectly.

## 6 Theoretical and practical implications

The study implications are multi-dimensional and offer both theoretical and practical implications. In present study, we developed a multi-dimensional, comprehensive model to assess students' acceptance and success of WLMS in the Indian educational setting. The multi-dimensional, comprehensive model is constructed based on different IS success models in an educational setting using the D&M IS success model as the base. It is assumed that the current model is comprehensive since various quality elements, including educational quality (which has not been studied earlier) user satisfaction, purpose to use, and benefits of using the WLMS, cover the critical components of the previous approaches.

Furthermore, research model explains the empirical evidence on critical factors that influence the WLMS success. In this study, four service quality elements are used as antecedents of user satisfaction, intention to use, and net benefits. Reliability and validity results show that all these elements are valid and essential in predicting WLMS success. The model fit statistics point to a good level of model fit that considers a novelty compared to previous studies. To the best of our knowledge, this is one of the few studies to provide a detailed description of the success factors for WLMS and to quantitatively analyse the relationships between the different measures in one single model in the Indian educational settings context. Finally, the research findings provide significant theoretical insights into the area of information system theories.

Many educational institutions in Tamil Nadu are now investing in the WLMS. However, WLMS implementation requires a huge amount of financial and other resources. Hence, findings of the study will help them to understand the success factors of WLMS and make an informed choice. Few educational institutions have already implemented the WLMS; our model will help them measure students' acceptance of the system and how successful it has been in meeting educational goals. Current study shows that students’ involvement moderates the success of a WLMS. Therefore, more initiatives must be taken to ensure their involvement in order to maximize the use of WLMS capabilities. To ensure involvement and acceptance, institutions must provide hands-on training to both students and instructors so as to familiarize them with all features and tools of the system and how they stand to benefit from using them.

## 7 Conclusion, limitations and future scope

The results confirm that the D&M IS serves as a useful model for understanding the factors that influence the WLMS success in an Indian educational setting. This study is the first empirical evaluation of the relevance of the D&M IS in predicting students' intentions, satisfaction and benefits of WLMS based on their academic involvement. It demonstrates that the D&M IS factors, such as technical system quality, information quality, educational system quality and service quality, positively influence student's intention to use the WLMS, satisfaction with the WLMS, and the net benefits.

The respondents were randomly selected from the Indian state of Tamil Nadu. The reliability and validity of the model can be further improved by selecting respondents with different demographical profiles from across the country. A comparative study between developed and developing countries will yield more insights. The focus of the current study is on students’ acceptance; future studies can extend the investigation to include different stakeholders, such as course administrators and instructors. The present study uses student academic involvement as a moderator; a study of other moderators such as learning style, online participation, perceived compatibility, and e-learning experience would provide greater insights. Present study is based on the D&M IS success model; however, future studies can consider other models, such as Technology Acceptance Model, Unified Theory of Acceptance and Use of Technology, and Theory of Planned Behaviour to develop more comprehensive models since ICT is a fast-evolving field.

## Declarations

### Author contribution statement

Subburaj Alagarsamy; Sangeeta Mehrolia; Indhu Sabari M: Conceived and designed the experiments; Performed the experiments; Analyzed and interpreted the data; Contributed reagents, materials, analysis tools or data; Wrote the paper.

### Funding statement

This research did not receive any specific grant from funding agencies in the public, commercial, or not-for-profit sectors.

### Data availability statement

Data will be made available on request.

### Declaration of interests statement

The authors declare no conflict of interest.

### Additional information

No additional information is available for this paper.
